# Utility of noninvasive biomarker testing and MRI to predict a prostate cancer diagnosis

**DOI:** 10.1007/s11255-023-03786-9

**Published:** 2023-09-24

**Authors:** Mark I. Sultan, Linda M. Huynh, Sarah Kamil, Ahmad Abdelaziz, Muhammed A. Hammad, Greg E. Gin, David I. Lee, Ramy F Youssef

**Affiliations:** Department of Urology, University of California, Irvine, Orange, CA USA

**Keywords:** Prostate biopsy, Prostate MRI, 4K score, ExoDx IntelliScore, Noninvasive prostate cancer testing

## Abstract

**Purpose:**

To assess the diagnostic performance and utility of the ExoDx IntelliScore and an OPKO4K score to predict prostate cancer in men presenting with elevated PSA—both as independent predictors and in combination with clinical/MRI characteristics.

**Methods:**

Patients with elevated PSA were retrospectively reviewed. Abnormal tests were defined as an OPKO4K score ≥ 7.5% and an ExoDx IntelliScore ≥ 15.6. Four regression models and ROC curves were generated based on: (1) age, PSA, and DRE, (2) model 1 + OPKO4K 4Kscore ≥ 7.5%, (3) model 2 + ExoDx IntelliScore ≥ 15.6, and (4) model 3 + MRI PIRADS 4–5.

**Results:**

359 men received an OPKO4K test, 307 had MRI and 113 had ExoDx tests. 163 men proceeded to prostate biopsy and 196 (55%) were saved from biopsy. Mean age was 65.0 ± 8.7 years and mean PSA was 7.1 ± 6.1 ng/mL. Positive biopsies were found in 84 (51.5%) men. The sensitivity and negative predictive value of an OPKO4K score were 86.7% and 72.3%; values for an ExoDx test were 76.5% and 77.1%, respectively. On regression analysis, clinical markers (Age, PSA, DRE) generated an AUC of 0.559. The addition of an OPKO4K score raised the AUC to 0.653. The stepwise addition of an ExoDx score raised the AUC to 0.766. The combined use of both biomarkers, patient characteristics, and MRI yielded an AUC of 0.825.

**Conclusion:**

This analysis demonstrates the high negative predictive value of both the OPKO4K score and ExoDX IntelliScore independently while demonstrating that the combination of an OPKO4K score, an ExoDX IntelliScore, and MRI increases predictive capability for biopsy confirmed prostate cancer.

## Introduction

Prostate cancer (PC) is the most common cancer among men with 73 cases diagnosed per 100,000 people, remaining the second leading cause of cancer deaths for males within the United States [1]. Early detection of PC is critical as the 5-year survival rate of localized PC is 99.9% compared to 29.8% in those with metastatic disease [2]. To detect occult PC, prostate specific antigen (PSA) was introduced in the early 1990s; however, PSA is non-specific for prostate cancer and cannot differentiate between aggressive or indolent tumors [3, 4]. This drawback has led to persistent overdiagnosis and overtreatment of low-risk PC, inciting the United States Preventive Services Task Force to confer a grade D recommendation for PC screening by PSA in 2012. Further, 22% of patients with a PSA level below standard screening guidelines will have detectable prostate cancer on biopsy, thus limiting the prognostic incite of established screening and potentially denying appropriate treatment for some patients [5].

PSA and digital-rectal exam (DRE) in combination persists as the current standard of care for noninvasive PC risk stratification. Though the decision to undergo a prostate biopsy remains of significant burden to both clinicians and patients. Unfortunately, many proceed to prostate biopsy unnecessarily—with a threshold of PSA > 4.0 ng/mL or abnormal DRE to determine the decision to biopsy, 68% of biopsies will yield no evidence of cancer [6]. Weighing against the risk of hematuria, rectal bleeding, urinary tract symptoms, infection, and sepsis [7], this low yield raises concerns regarding the potential harms and benefits in relying on an invasive biopsy to diagnose PC. To address the gap created by prostate biopsy overuse and its associated risks, various commercial biomarkers have been marketed to guide the decision for biopsy in men with an elevated PSA.

OPKO4K and ExoDx prostate cancer tests are among these noninvasive biomarkers [8–11]. OPKO4K is a blood test which assesses the risk of aggressive PC by combining four prostate-specific biomarkers (Total PSA, Free PSA, Intact PSA, and human Kallikrein 2 [hK2]) with important clinical factors (age, prior biopsy status, and optional DRE). Zappala et al. found OPKO4K to correctly identify 91.8% of men with high-risk PC, yielding a high positive predictive value in the risk assessment of aggressive PC regardless of DRE [10]. Similarly, ExoDx is a urine test designed to assess certain RNA biomarkers (PCA 3, ERG, SPDEF) to gauge a patient’s risk of aggressive PC. A 2019 study by Kohaar et al. demonstrated the ExoDx Prostate Cancer test significantly improved the predictive performance for diagnosing high grade PC compared to standard of care (*p* = 0.0009) while preventing 27% of unnecessary biopsies [9]. However, while both ExoDX and OPKO4k have been established to have high predictive capability independently, their combination in addition to imaging such as Magnetic Resonance Imaging (MRI) or other clinical characteristics has yet to be explored. In this regard, the present study seeks to assess the diagnostic performance of the ExoDx IntelliScore and OPKO4K score in predicting prostate cancer diagnosis in men presenting with elevated PSA—both as independent predictors and in combination with clinical/MRI characteristics.

## Patients and methods

### Patient population

From April 2017 to December 2019, 612 men presenting to a single center (University of California, Irvine Health) with an elevated PSA were retrospectively evaluated. Following institutional standard of care, all patients were offered MRI prostate imaging and biomarker testing, both an OPKO4K and an ExoDx test were offered simultaneously through an initial physician patient conversation regarding elevated PSA. Patients declining the OPKO4K test or those without adequate follow-up or further work-up, and those with a prior negative biopsy were excluded from analysis (*n* = 253). Of the 359 men with an OPKO4K test included in the analysis, 113 were additionally evaluated by an ExoDx prostate cancer test. Under an approved institutional review board protocol, results of both tests along with patient demographics, relevant clinical information, and biopsy results were retrospectively collected and entered into an electronic database. Data was de-identified and stored in compliance with the Health Insurance Portability and Accountability Act of 1996. All clinical, laboratory, and imaging data were discussed with each patient. Prostate biopsies were performed only in patients who agreed to proceed with biopsy after through discussion of all clinical, laboratory, and imaging data. Some patients elected PSA surveillance despite concerning PSA levels, biomarker test results, or MRI characteristics.

### Biomarker testing and imaging

#### OPKO4K

A venous phlebotomy sample for measurement was collected in a K2-EDTA tube for each consenting patient. Blood samples were obtained at the patient’s clinic visit and transported to the OPKO laboratory in Nashville, TN for analysis. The calculated probabilities were blinded from histopathology results and evaluated without DRE assessment. An abnormal test result was defined as an OPKO4K score ≥ 7.5%, as this has been validated to prevent 22% of unnecessary biopsies while maintaining a sensitivity of 97% and a negative predictive value of 99% for high grade disease [12].

#### ExoDx

For patients electing to also have an ExoDx Prostate Test performed following OPKO4K test results, a sample of 10–15 mL of first catch urine was requested from each patient as indicated by the ExoDx testing kit. Samples were stored at 4 °C for up to 5 days prior to transport to the Exosome Diagnostic laboratory in Waltham, MA for analysis. The output of the ExoDx test is a risk score (scale: 0–100), which predicts the presence of high-grade prostate cancer defined as greater than or equal to Gleason Group (GG) 2 upon biopsy. An ExoDx IntelliScore > 15.6 has been previously described and shown to discriminate biopsy-positive GG2 and above disease from GG1 and negative biopsies. A metanalysis of 1212 subjects with an IntellisScore cutoff of 15.6 has been validated to prevent 30% of benign and GG1 biopsies while maintaining a negative predictive value of 90% [13].

#### MRI

**Fig. 1:**
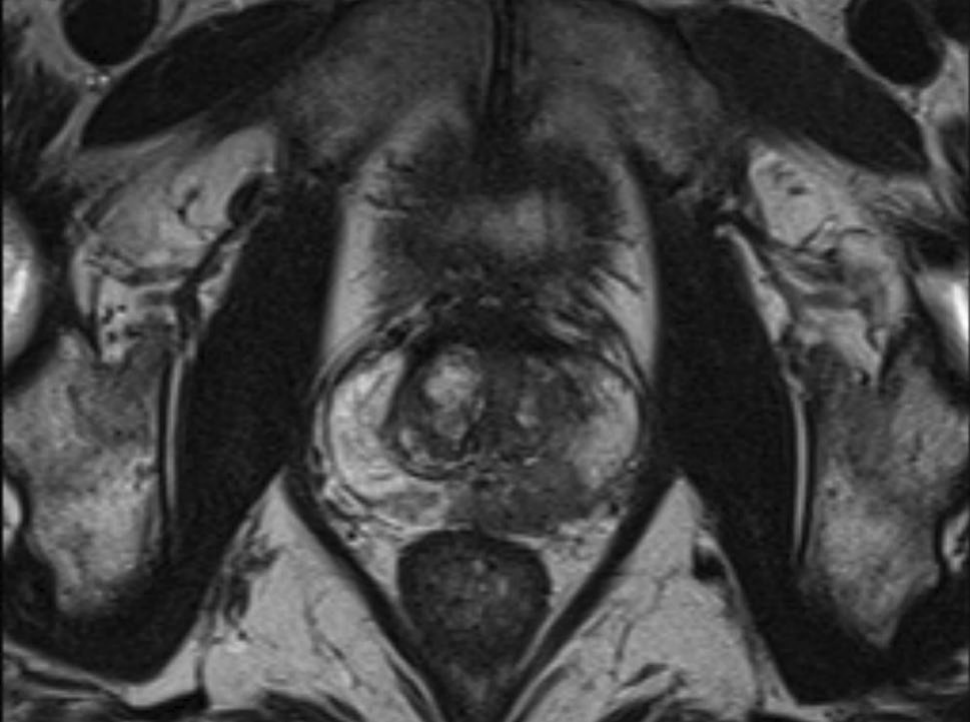
An axial T2-weighted image on a 3 T prostate MRI demonstrating a PI-RADS 5 lesion in the left peripheral mid gland

All MRI scans were interpreted by fellowship trained abdominal radiologists. MRI staging was defined by review of the radiologist rendered report, including the extent of prostate lobe involvement, extracapsular extension, and seminal vesicle involvement. All interpreting radiologists subjectively evaluated the multiparametric MRI (MP-MRI) scans and interpreted local invasive features based on the Prostate Imaging-Reporting and Data System (PI-RADS) v. 2.1 criteria [14]. Lesions rated PI-RADS ≥ 4 were considered a positive MRI result (Fig. 1).

### Outcome measures

**Fig. 2:**
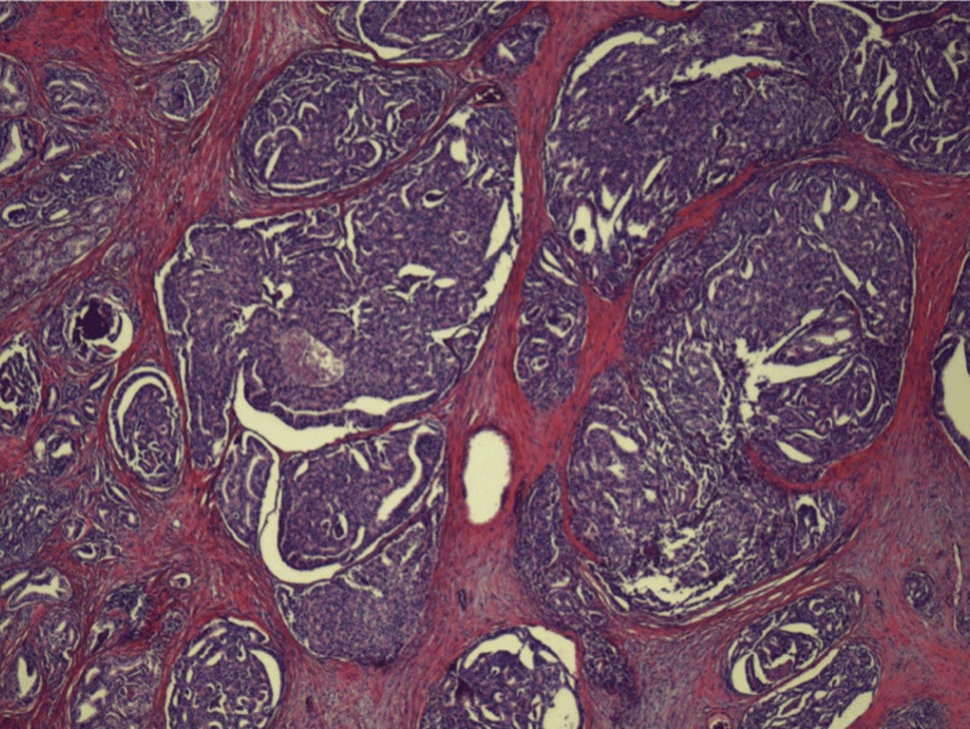
H + E low power view of patient region of interest biopsy demonstrating Gleason score 4 + 5 (GG5) prostate adenocarcinoma

Biopsies were done in a standardized fashion at a single institution. As such, number of biopsy cores, biopsy core areas, and pathologists were uniform for all patients included in the present analysis. This included a 12 core biopsy template as well as 2–3 cores from each region of interest present on prostate MRI (Fig. 2). The primary outcome was the diagnostic accuracy for PC, therefore, we assessed the sensitivity, specificity, negative predictive value, and positive predictive value of the OPKO4K and ExoDx tests to predict a positive prostate biopsy defined as GG 1 or greater. The secondary outcome measure was to assess the added clinical utility of the OPKO4K and ExoDx tests, in addition to patient characteristics including age, baseline PSA, DRE results, and MRI results.

### Statistical analysis

All analyses were performed using SPSS software version 25 (IBM Corp. Armonk, NY). Patient demographics and baseline clinical characteristics were summarized utilizing descriptive statistics, such that categorical variables were reported with n and %, while continuous variables were reported with mean and standard deviation (SD). Univariate associations between patient characteristics and positive prostate biopsy were assessed via Student t-test and regression modeling. Two-by-two contingency tables were generated to calculate unadjusted sensitivity, specificity, negative predictive values, and positive predictive values for both the OPKO4K and ExoDx tests independently.

Variables with a *p* value < 0.15 on univariate analysis were included in final regression modeling. Four logistic regression models were generated to predict probability of positive prostate biopsy; these included (1) patient age, baseline PSA, and DRE (2) model 1 + OPKO4K score ≥ 7.5%, (3) model 2 + ExoDx PC risk score ≥ 15.6, and (4) model 3 + MRI results. A *p* value less than 0.05 was considered statistically significant and 95% confidence intervals (95% CI) were reported for all odds ratios. From a logistic regression model, predicted probabilities of positive prostate biopsies were calculated for each patient with each of the four models and receiver-operator characteristic (ROC) curves were generated. Area under the curve (AUC) was calculated for each ROC curve and an asymptotic 95% confidence interval was reported for each area.

## Results

### Patient characteristics

Of the 359 included patients, the mean age at the time of initial presentation was 65.0 ± 8.7 years and the mean baseline PSA was 7.1 ± 6.1 ng/mL. Of these 359 men with OPKO4K test results, 113 (31.5%) also had ExoDx tests ordered. On univariate analysis, there were no significant differences in patient demographics or clinical characteristics (Age, PSA, DRE) for patients receiving only the OPKO4K test versus those receiving both the OPKO4K and ExoDx test.

Of the 359 patients with an OPKO4K test ordered, 272 (75.7%) resulted in a 4Kscore ≥ 7.5% and thus at risk for PC at subsequent biopsy. Of these patients, 163 (60%) proceeded to prostate biopsy and 84 (51.5%) resulted in a positive biopsy. Based on a positive biopsy result, the sensitivity, specificity, positive predictive value, and negative predictive value for a 4Kscore ≥ 7.5% was 86.7%, 69.6%, 52.1%, and 72.3%, respectively.

Of the 359 patients with OPKO4k test, 113 (31.5%) also had an ExoDx test ordered. Of those patients, 78 (69%) yielded a positive test defined as an ExoDx IntelliScore ≥ 15.6%. Based on subsequent positive biopsy results, sensitivity, specificity, positive predictive value, and negative predictive value for an ExoDx IntelliScore ≥ 15.6% were 76.5%, 65.8%, 33.3%, and 77.1%, respectively.

Finally, 307 patients (85.5%) had an MRI ordered. MRI results revealed 43 (14%) of patients had no appreciable lesion, 3 (1.0%) noted a PIRADS 2 lesion, 112 (36.4%) with a PIRADS 3 lesion, and 149 (48.5%) had a PIRADS 4 or 5 lesion.

### Multivariate modeling: clinical characteristics plus OPKO4K score

**Fig. 3:**
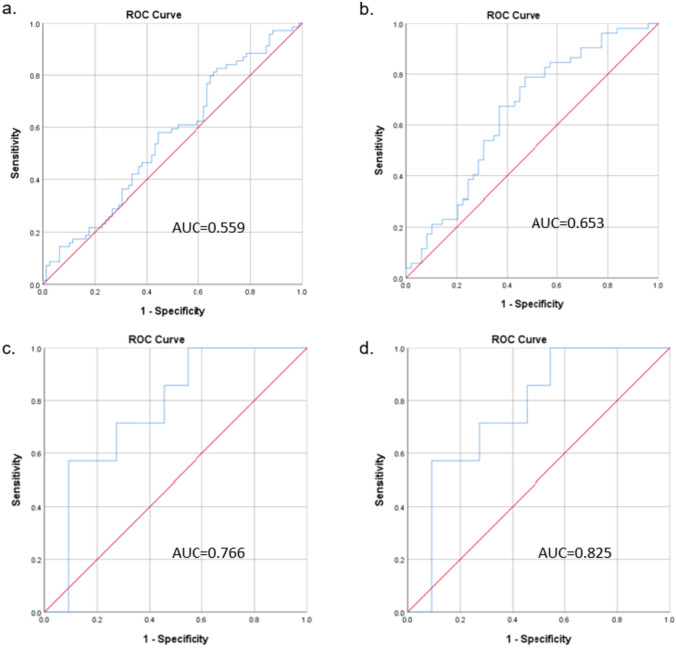
ROC curve based on a positive biopsy endpoint of **a** baseline patient characteristics, **b** baseline patient characteristics and the addition of OPKO4K Score, **c** baseline patient characteristics, OPKO4K score and the addition of the ExoDX Prostate Cancer Test, and **d** baseline patient characteristics, OPKO 4K score, ExoDX Prostate Cancer Test and MP-MRI results

Table 1 depicts a logistic regression model for standard of care patient characteristics to predict a positive prostate biopsy. The variables of patient age, baseline PSA, and DRE results were not significantly correlated with a positive biopsy result and this model yielded an AUC of 0.559 [0.466–0.651] (Fig. 3a). When the OPKO4K score was added to this baseline model (Table 2), a 4K score ≥ 7.5% was significantly correlated with positive biopsy in regression modeling (*p* = 0.018). The addition of OPKO4K increased the AUC to 0.653 [0.544–0.761] (Fig. 3b).

**Table 1:** Logistic regression model of patient characteristics (Age, PSA, DRE) for predicting a positive biopsy

	*B*	S.E	Wald	*df*	*p*	OR	95% CI for OR	
							Lower	Upper
Age, years	0.013	0.021	0.388	1	0.534	1.013	0.973	1.055
Baseline PSA, cont	0.016	0.028	0.329	1	0.566	1.016	0.961	1.075
DRE, negative [ref] vs positive	−0.579	0.373	2.410	1	0.121	0.561	0.270	1.164
Constant	0.902	1.305	0.478	1	0.489	0.406

**Table 2:** Logistic regression model of patient characteristics and OPKO4K score ≥ 7.5% to predict positive biopsy

	*B*	S.E	Wald	*df*	*p*	OR	95% CI for OR	
							Lower	Upper
Age, years	0.026	0.028	0.861	1	0.353	1.026	0.972	1.083
Baseline PSA, cont	− 0.045	0.036	1.550	1	0.213	0.956	0.892	1.026
DRE, negative [ref] vs positive	− 0.069	0.501	0.019	1	0.891	0.934	0.350	2.491
OPKO4K, negative [ref] vs positive	1.289	0.544	5.621	1	**0.018**	3.628	1.250	10.530
Constant	− 2.282	1.780	1.644	1	0.200	0.102		

A signficance of 0.018 demonstrates that an OPKO 4k score > 7.5% appeared to be the most signficant predictor of this logistic regression model which also included Age, baseline PSA, and DRE status (in bold)

### Multivariate modeling—adding ExoDx prostate test and MP-MRI

Table 3 depicts the addition of an ExoDX Prostate Test IntelliScore ≥ 15.6 to the previous model utilizing patient characteristics and OPKO4K score. The AUC improved to 0.766 [0.540–0.992] (Fig. 3c). Table 4 includes the addition of MP-MRI results to the model including standard of care screening, OPKO 4K score, and ExoDX Prostate Test. On a ROC curve generated to predict a positive prostate biopsy, the AUC improved to 0.825 [0.585–1.00] (Fig. 3d). In the summative logistic regression model, neither OPKO4K score ≥ 7.5%, ExoDx IntelliScore ≥ 15.6, nor MRI results alone were independent predictors of positive biopsy.

**Table 3:** Logistic regression model of patient characteristics, OPKO4K score ≥ 7.5%, and ExoDx IntelliScore ≥ 15.6 in predicting positive biopsy

	*B*	S.E	Wald	*df*	*p*	OR	95% CI for OR	
							Lower	Upper
Age, years	0.001	0.103	0.000	1	0.994	1.001	0.818	1.224
Baseline PSA, cont	− 0.363	0.318	1.305	1	0.253	0.696	0.373	1.296
DRE, negative [ref] vs positive	0.942	2.215	0.181	1	0.671	2.565	0.033	197.078
OPKO4K, negative [ref] vs positive	1.340	1.545	0.752	1	0.386	3.818	0.185	78.860
ExoDx, negative [ref] vs positive	1.149	1.278	0.809	1	0.369	3.156	0.258	38.654
Constant	−0.477	6.338	0.006	1	0.940	0.620

**Table 4:** Logistic regression model of patient characteristics, OPKO4K score ≥ 7.5%, ExoDx IntelliScore ≥ 15.6, and MRI results in predicting positive biopsy

	*B*	S.E	Wald	*df*	*p*	OR	95% CI for OR	
							Lower	Upper
Age, years	0.166	0.171	0.944	1	0.331	1.181	0.845	1.651
Baseline PSA, cont	− 0.135	0.414	0.106	1	0.744	0.874	0.388	1.966
DRE, negative [ref] vs positive	2.216	3.282	0.456	1	0.500	9.167	0.015	5695.716
OPKO4K, negative [ref] vs positive	0.186	1.977	0.009	1	0.925	1.204	0.025	57.966
ExoDx, negative [ref] vs positive	0.279	1.514	0.034	1	0.854	1.322	0.068	25.723
MRI, negative [ref] vs positive	2.908	1.733	2.818	1	0.093	18.325	0.614	546.727
Constant	–12.283	11.112	1.222	1	0.269	0.000		

## Discussion

The present study is a retrospective review directly assessing the diagnostic performance and clinical utility of ExoDx IntelliScore and OPKO4K score in predicting a positive biopsy in men presenting with an elevated PSA. Not only did the addition of OPKO4K to baseline patient characteristics for standard of care screening significantly increase predictive capability, but the combined use of ExoDx, OPKO4K, and MRI findings yielded improved discrimination between men with a low versus high probability of positive prostate biopsy, from an AUC of 0.559 to an AUC of 0.825. These findings significantly add to current risk stratification methods for men presenting with an elevated PSA and may potentially lead to fewer unnecessary prostate biopsies while maintaining clinical accuracy to discern high risk prostate cancer.

The authors of this manuscript are motivated to improve the diagnostic accuracy for PC with additional noninvasive testing as current standard of care relying on PSA is imperfect with limited specificity and escalating directly to prostate biopsy may not be well tolerated by some patients. Due to the overlap in elevated PSA levels for men with benign prostatic hyperplasia or chronic prostatitis, only 30–40% of patients biopsied on grounds of elevated PSA alone are diagnosed with prostate cancer [15]. In addition, there is biologic variability in serum PSA related to differences in androgen concentrations, prostate manipulation, and recent ejaculation [16]. For example, a study of 84 men noted a PSA mean variation of 15% within two weeks of sequential serum testing [17]. A published review of 2607 men who underwent transrectal ultrasound guided prostate biopsy revealed 4.0% of patients developed infectious complications including fever, urinary tract infection, acute prostatitis, epididymo-orchitis and sepsis within 7 days of biopsy [18]. Attributed to the rise in fluroquinolone resistant organisms, a Canadian analysis of 75,000 patients reported the risk of hospitalization after transrectal biopsy to be 1.9% [19]. These preventable hospitalizations remain a significant financial burden on health care systems, thus noninvasive testing holds potential to ameliorate these unnecessary costs.

First, we highlight the independent clinical utility of the OPKO4k score and ExoDx IntelliScore in predicting positive prostate biopsy. In the present study, an OPKO4K score ≥ 7.5% yields high sensitivity, specificity, and negative predictive value in predicting a positive prostate biopsy. Similarly, the use of ExoDx IntelliScore ≥ 15.6% yielded high sensitivity, specificity, and negative predictive value. These results align well with those of a 2017 meta-analysis by Zappala et al. [11] and a 2020 pooled analysis by Margolis and colleagues [13]. The high sensitivity and negative predictive value for both tests suit their purpose: the identification of benign elevations in PSA or clinically indolent disease. In other words, a negative result (i.e. an OPKO4K score < 7.5% or an ExoDx IntelliScore < 15.6%) rules out the presence of high-risk disease characteristics, ultimately prompting the avoidance of prostate biopsy and its associated risk for adverse events such as hematuria, rectal bleeding, infection, etc. A previous risk stratification model extrapolating data from 725 patients with a serum PSA between 4.0 and 10.0 noted an AUC of 0.53 on an ROC to predict any prostate cancer, our summative model improves predictive capability to an AUC of 0.825 [19]. These results parallel a 15-year longitudinal study following 12,542 Swedish men, when accounting for a PSA > 2 ng/mL at age 50 and an OPKO4k score > 7.5%, the predictive AUC for high risk disease was 0.863 [20].

Second, combining both the OPKO4K score and ExoDx IntelliScore significantly improved predictive capability when compared to standard patient characteristics alone or with independent use of either test. When combining both tests in conjunction with standard screening (Age, DRE, PSA) and MRI results, the AUC improved significantly from 0.559 to 0.825. This improvement in predictive capability translates well to the clinical setting—in essence, allowing the clinician to rule out patients with a high probability of clinically indolent disease or benign rises in PSA such as benign prostatic hyperplasia or chronic prostatitis. The inclusion of ExoDx is helpful—without the addition of this second biomarker, the AUC would have been 0.653 rather than 0.766. This stepwise improvement is similar to the results of a study by Nordstrom et al. comparing the combined predictive value of an OPKO4K test with the Prostate Health Index (PHI). While both tests independently out-performed PSA alone, the combined use of both biomarker panels significantly reduced the number of unnecessary prostate biopsies than when used independently [21].

Overall, these findings corroborate the added benefit of the OPKO4K and ExoDx biomarker tests in the decision to biopsy. When used in conjunction with traditional factors such as age, baseline PSA, and DRE, the addition of ExoDx Prostate Test and an OPKO4k score significantly improved the ability to predict patients at high-risk for PC and (more importantly), to exclude patients with high probability of clinically undetectable or indolent disease. Even further, utilizing both the OPKO4K and ExoDx tests prior to MRI provides outstanding discrimination between men with a low versus high probability of positive prostate biopsy. Thus, providing the potential to decrease the number of unnecessary prostate biopsies with its associated adverse effects, particularly for patients with a low risk of PC.

Considering these results within limitations of the study context, this analysis represents a single institution retrospective analysis and may be limited by sample size and influenced by referral patterns. In addition, pathologic results require the integration of many elements associated with a biopsy such as the number of biopsy cores, immunohistochemical stain performance, and pathologist expertise. However pathological evaluation was done with a GU cancer pathologist in an academic center and these factors were considered part of our clinical practice; particularly when discussing management after positive biopsy. Another consideration ought to be toward the cost impact of multiple noninvasive tests, given our southern California patient demographic, these tests have not demonstrated to be cost prohibitive to patients. In addition, a 2018 United States cost-effectiveness analysis demonstrated both 4K and ExoDx tests to be cost-effective strategies independently, however, the model did not account for more than one biomarker test [22]. Furthermore, we had more patients with an OPKO4K Score and a MP-MRI than ExoDx Prostate test, this was a consequence of patient choice. While this may have resulted in a smaller number of ExoDx tests ordered, this decision-making process represents true clinical practice. Regardless, the varied number of patients receiving biomarker testing, MRI, and even prostate biopsies suggests a need for a multi-institutional prospective trial prior to broad application of these findings. Further investigation into the role of novel, commercially available biomarkers is warranted with a particular focus on the variation of longitudinal results for both high and low-risk patient populations in addition to the value of biomarker combination and their utilization with MP-MRI. Although the present study seeks to address prostate cancer diagnosis, future efforts will apply these biomarkers to detect clinically significant prostate cancer (i.e., GG2 and above). Even further, combination with post-diagnostic risk scores such as the Decipher test or GenomeDx risk scores could enhance PC management toward decreasing post-treatment recurrence rates, probability of distant metastases, and ultimately aim to reduce prostate cancer specific mortality.

## Conclusion

This study demonstrates the value of the OPKO4K score and the ExoDX IntelliScore—both performing well independently with high negative predictive values in the decision making paradigm to proceed with prostate biopsy. The addition of the ExoDx Prostate test, an OPKO4K score, and MP-MRI to standard screening which includes age, baseline PSA, and DRE displayed excellent predictive diagnostic performance. However, given the overlap in the confidence intervals of our various models, these results remain hypothesis generating and warrant a larger analysis. Overall, these findings represent an opportunity to further integrate the use of these novel biomarkers and their combination with MRI into clinical practice and future clinical trials with hopes to limit the frequency of unnecessary prostate biopsies while clinically discerning high risk localized disease.

## Data Availability

The data was generated by UC Irvine department of Urology. Though these data are not available online, the authors are prepared to share data with the editors upon reasonable request.

## Abbreviations

PC: Prostate cancer
PSA: Prostate specific antigen
DRE: Digital rectal exam
MP-MRI: Multiparametric magnetic resonance imaging
GG: Gleason group
PI-RADS: Prostate imaging-reporting and data system
ROC: Receiver operating characteristics
AUC: Area under curve
PHI: Prostate health index
